# Barriers to accessing internet-based home Care for Older Patients: a qualitative study

**DOI:** 10.1186/s12877-021-02474-6

**Published:** 2021-10-18

**Authors:** Baosheng Zhao, Xiaoman Zhang, Rendong Huang, Mo Yi, Xiaofei Dong, Zhenxiang Li

**Affiliations:** 1 Shandong Provincial Hospital, Cheeloo College of Medicine, Shandong University, No. 324 Jingwu Weiqi Road, Jinan, Shandong Province 250021 China; 2 Shandong Provincial Hospital Affiliated to Shandong First Medical University, No. 324 Jingwu Weiqi Road, Jinan, 250021 Shandong Province China; 3 School of Nursing, Hangzhou Medical College, No. 8 Yikang Road, Hangzhou, Zhejiang Province 311399 China; 4 School of Nursing and Rehabilitation Shandong University, No. 44 Wenhuaxi Road, Jinan, Shandong Province 250012 China

**Keywords:** Internet, Home care, Online nurses, Older patients, Barriers, Qualitative study

## Abstract

**Background:**

Due to the increasingly ageing society and the shortage of nursing human resources in China, the imbalance between the home care needs of older patients and the inadequate supply of nursing services is increasing. Based on this medical situation, China is implementing internet-based home care (with the nurses who provide this care called online nurses or sharing nurses) based on the concept of the sharing economy, internet technology and knowledge from the home care experience in other countries. Internet-based home care follows an online application/offline service model. Patients place orders through an app, nurses grab orders instantly, and managers dispatch orders through a web platform based on various factors such as nurses’ qualifications, professionalism and distance from the patient. In this way, home care is provided for patients with limited mobility, such as older or disabled patients, patients in rehabilitation and terminal patients. Only by fully understanding the barriers to accessing internet-based home care can we provide quality nursing services to older patients and achieve the sustainable development of internet-based home care.

**Objective:**

The goal of this study was to use qualitative methods to explore barriers to accessing internet-based home care for older patients.

**Methods:**

Based on Levesque’s access to health care framework, semi-structured personal interviews were conducted with 19 older patients in a descriptive qualitative study using directed content analysis.

**Results:**

We identified four barriers to accessing internet-based home care for older patients: barriers to perceiving, seeking, paying for, and engaging in internet-based home care. Specific barriers included traditional perceptions, barriers to internet use, high payment costs, uneven quality of services, and concerns about privacy and patient safety.

**Conclusions:**

Internet-based home care brings new risks and challenges. In order to enable older patients to better enjoy it, it is necessary to strengthen publicity, optimize the network application process, improve the health insurance system, formulate unified nursing service standards, and address safety risks.

**Supplementary Information:**

The online version contains supplementary material available at 10.1186/s12877-021-02474-6.

## Introduction

According to the National Bureau of Statistics, by the end of 2017, the number of people aged 60 and above in China was 240 million, with those aged 60 and above accounting for 17.3% of the total population. In addition, 150 million older people suffer from chronic diseases, accounting for 65% of the total older population, and approximately 40 million older people are disabled or semi-disabled, leading to a rapidly growing demand for home care [1, 2]. Home care has become an effective way to cope with ageing in the United States, Canada, Japan and other countries [3–6]. Home care in China is usually provided by community nurses, but due to the inadequate level of community care and human resources, it is not yet possible to meet the home care needs of the public [7, 8].

The sharing economy refers to a system in which an organization or individual who owns idle resources transferring the right to use the resources to others for a fee; the transferor receives a return, and the sharer creates value by sharing others’ idle resources [9]. Based on this concept, China launched internet-based home care (with the nurses who provide such care being called online nurses or sharing nurses). Internet-based home care is provided by registered nurses from fixed medical institutions, and an online application/offline service model is implemented. Patients place orders through an app, online nurses use idle off-duty time to grab orders instantly, and managers dispatch orders through a web platform based on various factors such as online nurses’ qualifications, professionalism and distance. In this way, home care is provided for people with limited mobility, such as older or disabled patients, patients in rehabilitation and terminal patients [10]. Currently, through patients’ online applications, nurses grab orders from the app, and management dispatches orders, nurses travel to patients’ homes to provide services including routine nursing operations such as intramuscular injections, intravenous injections, urinary catheterization, gastric tube insertion and blood sample collection, as well as specialty care such as Peripherally Inserted Central Venous Catheters (PICC) medication exchange, wound stoma care and neonatal examinations [11].

Internet-based health services mainly use the internet medium for telemedicine intervention or supervision [12–14]. China applies internet to home care, as a new service model in which nurses provide nursing care at patients’ homes after grabbing orders via the Internet, Internet-based home care can maximize nurse human resources, meet home care needs, increase nurse salaries, improve nurse professional recognition, etc., but it also increases the new risks and challenges of home care [10, 11]. Both internet-based home care in China and home care in other countries involve nurses coming to patients’ homes to provide home care. The difference between them is that, first, home care in other countries mostly serves established patients, provides continuous nursing services, and creates a stable nurse-patient relationship [15, 16]; on the other hand, in internet-based home care, online nurses accept orders on-demand through a web platform, so each time they meet new patients, provide one-time home care, and must establish a new nurse-patient relationship. Second, home care in other countries is mostly employed by agencies specializing in home care services [17, 18], and nurses are mostly employed full time [19–21]. In contrast, In contrast, online nurses are affiliated with hospitals who provide home care on their idle off-duty time, and online nurses are part-time.

For older patients to universally accept and participate in internet-based home care, it is first necessary to understand the factors that may prevent them from using internet-based home care. Therefore, the purpose of this study was to understand what factors prevent older patients from using internet-based home care. Although previous studies have described the barriers encountered in home care [22, 23], there are no studies of internet-based home care.

Levesque [24] argued that access to health care is multidimensional and includes five dimensions: 1) Approachability, 2) Acceptability, 3) Availability and accommodation, 4) Affordability, 5) Appropriateness, that barriers may be encountered in each dimension of access and that users must have the corresponding capabilities. Levesque identified five corollary dimensions of abilities: 1) ability to perceive, 2) ability to seek, 3) ability to reach, 4) ability to pay, and 5) ability to engage. According to the authors,the ability to perceive is determined by such factors such as health literacy, knowledge about health and beliefs related to health and sickness. The ability to seek health care relates to the concepts of personal autonomy and capacity to choose to seek care, knowledge about health care options and individual rights that would determine expressing the intention to obtain health care. The ability to reach health care relates to the notion of personal mobility and availability of transportation, and occupational flexibility that would enable one person to physically reach service providers. The ability to pay for health care is a widely used concept within the health services and health economics literature. It describes the capacity to generate economic resources - through income, savings, borrowing or loans - to pay for health care services without catastrophic expenditure of resources required for basic necessities. The ability to engage in health care would relate to the participation and involvement of the client in decision-making and treatment decisions, which is in turn strongly determined by capacity and motivation to participate in care and commit to its completion. This dimension is strongly related to the capacity to communicate as well as notions of health literacy, self-efficacy and self-management.

## Methods

### Design

The study used a qualitative design, and data were collected through semi-structured personal interviews. We obtained approval from the ethics committee of Shandong Provincial Hospital Affiliated to Shandong University. (NO.2016–130).

### Sampling and recruitment

The study was conducted in Jinan City, Shandong Province, China. We recruited older patients who were older than 60 years old, were in stable health (exclude older patients with acute and critical illnesses and those whose communication aggravates their condition), and had normal verbal communication skills (exclude older patients with aphasia, extubation, tracheotomy, etc. who communicate physically or with the aid of devices). To ensure the collection a wide range of information, we used the maximum variation strategy to select patients who had placed orders on the online information platform and received internet-based home care services based on their characteristics such as age, gender, education level, type of household, and type of service. In addition, according to the conditions and needs of the older patients, we match older patients who are suitable for internet-based home care but always choose hospital visits.

### Data collection procedure

The literature review was designed to provide a systematic understanding of relevant research in order to outline a comprehensive and concise, appropriate interview and an interview outline was initially prepared after thorough discussion among the research team. Two patients were selected for pre-interviews, and the formal interview outline was revised again according to the interview results, as shown in Appendix 1.

All interviews were conducted between November 2020 and April 2021, and the sample size was determined based on the point when data saturation was reached in the interviews. The older patients who had received internet-based home care had limited functionality and limited mobility. We conducted semi-structured interviews with the 13 elderly patients in their homes. For the 7 matched hospital patients, the semi-structured interviews were conducted in hospital wards or outpatient lounges.

Semi-structured interviews were used to collect data, and the interview times were agreed upon with the respondents in advance. The researcher established good rapport with the interviewees through the hospital and online platforms in advance, explained the purpose and significance of the study before the interview, and obtained signed informed consent forms from the respondents in which they consented to the interviews being recorded and other issues. During the interview process, the researcher listened carefully, maintained a neutral attitude, encouraged the interviewees to fully express themselves and their ideas, and observed and recorded the non-verbal communication of the interviewees. The interviews were conducted with the interview outline in mind, with appropriate follow-up questions, repetitions, and summaries, and each interview lasted 30 to 60 min. After each interview, the researcher kept a reflective journal to improve the quality of the interview analysis.

### Data analysis

Within 24 h of the interviews, the audio recordings were independently transcribed by two researchers, and the data were analysed simultaneously by two researchers,with disagreements or ambiguous concepts discussed as a group. Because this study used Levesque’s access to health care framework as its theoretical framework, the data collection was more structured than what is usual in many other qualitative studies, and the analysis process was more explicit [25, 26]. The analysis was conducted using the directed content analysis method [27, 28], which includes 3 stages: preparation, organization, and reporting. The specific steps were as follows. (1) The unit of analysis was defined, and the sentences that reflected the barriers to older patients’ access to internet-based home care were used as the smallest units of segmentation to form the unit of analysis. (2) The original data were reviewed and read repeatedly. (3) A classification outline was developed based on the model to determine the categories of the unit of analysis.(4) Content coding and categorization were performed; open coding and marking of important ideas and concepts in the data were carried out, and similar codes were grouped into corresponding categories to form themes and sub-themes. (5) The results were interpreted and analysed; links between the data and results were formed, and corresponding excerpt examples from the data were identified.

## Results

### Participants

Participants’ demographic characteristics are provided in Table 1. Of the 19 participants, approximately half (*n* = 11) were male, and all were older than 60 years, with an average age of 70 years (range 61 to 82). More than half of the participants had an education level of junior high school or below. More than half of the participants lived in urban communities, and the main types of care services received by the interviewed patients were: replacement of gastric tube, replacement of urinary catheter, PICC medication exchange, intramuscular injection, Intravenous blood collection,etc.

**Table 1:** Characteristics of the Participants

Number	Gender	Age	Educational Level	Household Registration	Main types of care services received by the interviewed patients
P1	Male	78	Primary school graduate	City residence	Replacement of gastric tube
P2	Male	69	Middle school graduate	City residence	Replacement of gastric and urinary catheters
P3	Female	72	Primary school graduate	Rural residence	PICC medication exchange
P4	Male	69	High school graduate	City residence	Intramuscular injection
P5	Female	62	College graduate	City residence	Intravenous blood collection
P6	Female	82	Middle school graduate	City residence	Geriatric companionship
P7	Male	68	High school graduate	City residence	Replacement of urinary catheter; pressure sore management
P8	Male	71	Middle school graduate	Rural residence	Replacement of gastric and urinary catheters
P9	Female	68	College graduate	City residence	Diabetic foot medication change
P10	Female	70	Middle school graduate	City residence	Stick moxibustion
P11	Female	61	High school graduate	City residence	Intravenous blood collection
P12	Male	63	Primary school graduate	Rural residence	Intramuscular injection
P13	Male	78	Primary school graduate	Rural residence	PICC medication exchange
P14	Male	65	High school graduate	City residence	Intramuscular injection
P15	Male	71	Middle school graduate	Rural residence	Stoma care
P16	Female	69	High school graduate	City residence	Replacement of gastric tube
P17	Female	73	Middle school graduate	Rural residence	PICC medication exchange
P18	Male	69	Primary school graduate	Rural residence	Intravenous blood collection
P19	Male	69	Middle school graduate	City residence	Replacement of urinary catheter

### Themes

Among the barriers to internet-based home care for older patients, the following four themes were identified: (1) barriers to perceiving internet-based home care; (2) barriers to seeking internet-based home care; (3) barriers to paying for internet-based home care; and (4) barriers to engaging in internet-based home care. See Table 2 for details.

**Table 2:** Themes and Subthemes

Themes	Subthemes
**Barriers to Perceiving Internet-based Home Care**	Cognitive bias; Traditional perceptions of access to care; Preference for care by children
**Barriers to Seeking Internet-based Home Care**	Insufficient access to information; Low use of the internet; Low trust in the internet; Declining physical function; Relatively cumbersome process; Children placing orders for them
**Barriers to Paying for Internet-based Home Care**	Expensive; Lack of standardization of fees; Lack of health insurance policy support
**Barriers to Engaging in Internet-based Home Care**	Inadequate integration and relevance; Limited service items; Limited service hours; Lack of service continuity; Untimely internet-mediated feedback; Leakage of personal information; Uneven service quality; Potential risks to patient safety

### Barriers to perceiving internet-based home care

Older patients’ cognition of internet-based home care is biased, such as the formality of the service, its legality, and the type of service recipients. They believe that internet home care lacks formality and is applicable only to patients with limited physical activity who are unable to take care of themselves and have chronic and serious illnesses.

*P14: "I am still physically strong and can move freely [ … ] When I get older and can't move, I might choose it."*

*P16: “The online nurse service is not as reliable as the hospital, and I don't know if it is formal and legal [ … ] I think only seriously ill patients would place orders, just like calling an ambulance."*

Due to years of medical perception and habits, older patients tend to adopt traditional coping methods, preferring familiar hospital environments and nurses.

*P17: "For each change in medication, I contact Nurse Xiao Liu (Pseudonym) in advance; she is more familiar with me. I come directly to the department, and she changes my medication, forming a habit."*

Older patients prefer that their children provide care for them and believe that online nurses do not have enough intimacy with older patients to replace the emotional support given by their children.

*P6: "My son used to accompany me on check-ups, and he has been too busy lately [ … ] Although the nurse is very responsible, I feel empty inside without my son's company."*

### Barriers to seeking internet-based home care

The older patients have limited access to information, mostly through traditional media such as newspapers and TV, and thus do not have sufficient access to information related to internet-based home care.

*P13: "I didn't know there were online nurses before, so I called 120 to go to the hospital to change my medicine, and the nurse told me that there was already online nurses. Since then, I've been placing orders online; it's so convenient."*

*P18: "I haven't heard of online nurses. I haven't heard my son mention it. It's not in the newspapers or on TV. I can't access this information."*

With the onset of age and disease, older patients experience a gradual decline in various bodily functions and often have reduced audio-visual function, which affects their use of the internet; for example, they face obstacles in finding web pages, reading text in different fonts, and changing volume levels.

*P5: "Due to presbyopia with age, the words on the phone are too small to see clearly; when I look at them a while, my eyes become blurry eyes and start to tear up [ … ] the application process is also more cumbersome."*

In addition, because older patients have less access to the internet, poor information technology and a low trust in the internet, they are unable to complete the internet-based home care ordering process, and most of them have their children place orders on their behalf.

*P1: "Through the online appointment system, only young people can operate it; the older cannot operate it. Every time, I ask my son to make an appointment[ … ] you can talk to him."*

*P19: "I have less contact with the internet. I feel unsafe. What if I meet a fraudster online? On the other hand, a hospital visit is safe."*

### Barriers to paying for internet-based home care

The cost of internet-based home care is relatively expensive, so ordinary families consider it carefully. In addition, there is a lack of standardization of fees, and older patients worry about indiscriminate charges.

*P9: "Although internet-based home care provides convenience [ … ] the price charged is expensive, more than 10 times the cost of going to the hospital. It would be good if it could be cheaper."*

*P3: "At that time, my son downloaded 2 apps, and the fees for each are different; both are quite expensive. What about indiscriminate charges?"*

At present, services are paid for by patients themselves and are not yet covered by medical insurance, resulting in a heavy financial burden for patients.

*P9: "Currently all services are paid out-of-pocket and are not reimbursable; having the health insurance pay earlier would be better.”*

*P11: "A lot of hospital visits can be reimbursed by medical insurance[ … ] if internet-based home care could also be included in medical insurance, plus the savings in transportation costs, registration fees, and no need to go to the hospital, it could be very popular."*

### Barriers to engaging in internet-based home care

At present, internet-based home care is not sufficiently holistic and does not provide appropriate levels of interaction, and the services are limited and administered individually, ignoring the relatedness of chronic diseases and other diseases. In addition, the service hours are generally limited to the daytime, which makes it difficult to meet the diverse needs of patients.

*P2: "You may need to place separate orders to change gastric and urinary catheters, and you want to receive infusions at home, but currently there is no such option for placing orders [ … ] so I hope that in the future, you will be able to place orders based on diseases and include a variety of care services rather than being limited to a certain operation."*

*P4: "Generally, you have to place orders during the day; no one takes orders at night. That is very annoying. Once, I placed an order at night, and the nurse said her home was nearby [so she would complete the visit], but she said that she generally does not take orders so late."*

Because internet-based home care is provided as a single, transient service, the continuity of nursing care is not sufficient. When the nurse leaves the patient’s home, it is difficult to deal with other nursing needs or adverse reactions. Although each information platform has a “question consultation” or “online chat” function, the feedback from patients after consultation is not timely due to network delays, a lack of fixed personnel and the business of clinical work.

*P8: "The nurse leaves after the service, unlike in the hospital where you can always ask the nurse if you have questions"*

*P7: "Although you can communicate with the nurses through cell phones, there may be delays in communicating over the internet; plus, the nurses are busy and may not look at their phones in time, so the feedback is not very timely."*

Older patients are concerned about personal information being leaked. This is especially true in regard to personal information sharing and the online nurses’ use of video recording during their work so that the entire process can be traced.

*P11: "When applying you need to fill in personal information, service needs and information about your illness; all nurses are able to see my information [ … ] if the information were to be used by unscrupulous people, the consequences would be serious."*

*P8: "Video recordings are taken during the procedures. I feel very uncomfortable; it is a bit of an invasion of my privacy [ … ] I had raised comments, and the nurse said the platform requires it and did not look into it deeper."*

Older patients are highly concerned about safety issues and have concerns and worries about the quality and efficiency of internet-based home care and the quality of nurses. In addition, it is difficult for the home environment to meet the same requirements of the hospital environment, and the lack of timely assistance from others in case of operation failure and the lack of timely support from a medical team in handling emergencies leads to a lack of patient safety.

*P10: "The level of skill of each nurse is different; I remember a nurse who liked to smile and make people feel warm, and she was also skilled and worked more smoothly than others [ … ] it would be good if every nurse could do it [like her]."*

*P1: "For a long time need, my gastric tube needed to be changed to my other nostril. The nurse tried several times without success and later found an old nurse, and it was finally solved [ … ] a lot suffering, and the experience was very bad."*

*P4: "There is a big difference between home and hospital [ … ] if there is an unexpected situation, it is difficult for the nurse to handle it alone, and safety is not guaranteed."*

## Discussion

### Principal findings

In this paper, we explored the barriers to accessing internet-based home care for older patients using Levesque’s access to health care framework and identified four barriers to home care for older patients: barriers to perceiving internet-based home care, barriers to perceiving internet-based home care, barriers to paying for internet-based home care and barriers to engaging in internet-based home care. According to Levesque’s access to care framework [24], which concerns routine patient hospital visits, patients do not need to arrive at the hospital to receive internet-based home care, and no specific barriers are present in reaching internet-based home care.

Older patients’ traditional perceptions of medical care and cognitive biases about internet-based home care have a significant impact on their perceptions of internet-based home care. The subjective perceptions of older patients that internet-based home care lacks formality and is mostly for seriously ill patients, coupled with the fact that older people are less likely to use the internet as a communication tool for health-related matters [29, 30], has led to low acceptance of the new industry of internet-based home care by older patients. Only by strengthening comprehensive health education and making older patients correctly understand the connotation and importance of internet-based home care can we improve their sense of identification with the service and stimulate their potential demand. In addition, because internet-based home care cannot replace the emotional support provided by children, older patients tend to prefer their children’s care, which not only increases their children’s caregiving burden [31, 32] but also may negatively affect the physical health of older patients due to their children’s low professional quality [33, 34].

Currently, information is mostly sought through online media, but online social media is not widely used among older patients [35], which, combined with older patients’ own limited internet technology and low trust in the internet, leads to insufficient access to medical information for older patients. It is imperative to enhance the publicity of internet-based home care in a way that is appealing to older patients and to make more older patients aware of online nurses. In addition, with age and disease, older patients’ bodily functions, such as memory, vision, and sense of touch, gradually decline, resulting in significant barriers to their use of network devices and mobile smart apps, such as their ability to perform webpage searches, read text in different fonts, and change the volume leve [36]. The operating interface of smart apps should be simplified according to the feedback of older patients to improve the experience of older patients. Another important finding is that older patients often share control and decision-making power over internet-based home care with family members to help cope with this complex situation [37, 38]. This tendency can facilitate the application of internet-based home care in the older patient population.

The high cost of internet-based home care is an important factor that hinders its promotion. Because factors such as transportation cost and time cost must be considered in internet-based home care, the cost is many times higher than that of regular hospital visits; there is no uniform standard for service item charges; and most costs are determined by the platform according to the service item, service time and service distance [10]. At present, internet-based home care is paid completely out of pocket by patients and services are not reimbursed by medical insurance, so there is a contradiction between residents’ demand for services and their ability to pay. We can learn from Japan, Germany, and other ageing countries that incorporate home care into a separate long-term care insurance system [39, 40] or from the U.S. integrated care model in which Medicare pays for home care on a per capita basis [41]. In addition, we should fully consider the role of the market and build a price mechanism that is suitable for the local area to maximize the interests of both nurses and patients and ensure the accessibility of internet-based home care.

There is a gap between the current internet-based home care and the market demand in terms of service items, service time, and service continuity, and many improvements need to be made to form comprehensive, coordinated, and high-quality internet-based home care [42, 43]. In addition, most internet-based home care involves the use of video recordings to document the whole service process, and a balance between ensuring the safety of nursing patients and protecting patients’ personal privacy needs to be achieved [44, 45]. Finally, safety is a central issue in the home care of older patients [46, 47]. Currently, the quality of nursing services varies, and there is a risk of nurses having insufficiently accurate and complete patient information; moreover, nurses mostly provide care alone, and when nurses encounter changes in a patient’s condition or require emergency assistance, they may not be able to implement effective treatment due to their lack of personal competence, lack of prescribing authority, lack of emergency equipment, and lack of medical team support, thus endangering patients’ lives [48, 49]. In the future, we must also improve the training system, develop implementation rules and clear processes, establish a quality control system, and improve emergency access to ensure patient safety.

## Limitations

First, although we succeeded in collecting a diverse and disparate sample to reach information saturation, the participants had certain geographical limitations, all from Jinan, Shandong Province, and were underrepresented for other regions. Second, although participants who received Internet home care were matched with hospital participants, the differences in experience between them were not significant. Finally, we explored barriers from the perspective of older patients only, and further exploration from the perspective of younger patients, nurses, and caregivers is necessary.

## Conclusions

Internet-based home care brings new risks and challenges. In order to enable older patients to better enjoy it, it is necessary to strengthen publicity, optimize the network application process, improve the health insurance system, formulate unified nursing service standards, and address safety risks.

## Supplementary Information

**Additional file 1: Appendix 1.** Interview Outline.

## Data Availability

The results/data/figures in this manuscript have not been published elsewhere, nor are they under consideration by another publisher.
