# Supplementary material for: Barriers to accessing internet-based home Care for Older Patients: a qualitative study
Source: BMC Geriatr. 2021 Oct 18;21:565. doi: 10.1186/s12877-021-02474-6 (PMC8522081; doi:10.1186/s12877-021-02474-6)
Supplement: Supplementary file 1 — Additional file 1: Appendix 1. Interview Outline. [file 12877_2021_2474_MOESM1_ESM.docx]

**Appendix 1 Interview Outline**

**Interview outline 1: (Patients receiving internet-based home care)**

(1) How did you learn about online nurses? Can you tell us what you know about online nurses?

(2) Which online nursing services have you received? Did they meet your nursing needs?

(3) Why did you choose an online nurse? What are the shortcomings compared to hospital visits or family care?

(4) How did you apply for an online nurse? What barriers did you face in applying?

(5) How did you pay for the services? What barriers have you encountered in making payments?

(6) What are your experiences and feelings about receiving online nursing? Were there any issues that worried you?

(7) Besides the above, what do you think are the shortcomings or areas for the improvement of online nurses?

**Interview outline 2: (Patients receiving care in routine hospital visits)**

(1) What are the inconveniences of your hospital visits? Would you prefer to receive care at home?

(2) Are you aware of online nurses? Can you tell us about your understanding of online nurses?

(3) Would you choose an online nurse in the future? Can you tell us why?

(4) What services do you think online nurses should provide? Are there any other requirements?

(5) What do you think are the barriers to the vigorous implementation of online nursing?

**General probes**

(1) Can you explain this in detail?

(2) Can you think of an example?

(3) How do you feel about it?

(4) What do you suggest?

(5) Do you have anything else to add?
